# Reduction of a Luxation of the Humerus of Thirty One Days Standing

**Published:** 1838-02-14

**Authors:** T. Harris

**Affiliations:** Surgeon to the hospital


### CLINICAL REPORTS.

PENNSYLVANIA HOSPITAL.

*Reduction of a Luxation of the Humerus of thirty one days standing, by* T. Harris, M. D., *Surgeon to the hospital.*

This was a luxation forwards under the pectoral muscle; no previous attempt at reduction had been made. The man, a remarkably robust subject, 48 years of age, was admitted into the hospital Jan. 24th. On 27th, Dr. Harris, present also Drs. Randolph and Norris, succeeded in restoring the head of the bone to its natural situation. At the commencement of the operation, the man was bled ℔℥xxx, and took in all three grains of tartar emetic. Extension was kept up downwards and backwards by the pulleys for twenty minutes, at the end of which time, the head of the bone was pressed downwards by the knee, and slipped into the axilla. The man was then laid on the floor, the heel placed in the axilla, and two assistants drew the arm downwards, and, at the end of five minutes, the bone went into its place with an audible snap. Clavicle bandage applied, and the man discharged same afternoon, at his own request. The operation lasted twenty five minutes.

